# Dopamine response gene pathways in dorsal striatum MSNs from a gene expression viewpoint: cAMP-mediated gene networks

**DOI:** 10.1186/s12868-020-00560-w

**Published:** 2020-03-26

**Authors:** Vladimir N. Babenko, Anna G. Galyamina, Igor B. Rogozin, Dmitry A. Smagin, Natalia N. Kudryavtseva

**Affiliations:** 1 Institute of Cytology and Genetics SB RAS, Novosibirsk, Russia; 2 Novosibirsk State University, Novosibirsk, Russia; 3 National Institutes of Health, Rockville Pike, Bethesda, MD USA

**Keywords:** Dorsal striatum, Mouse model of chronic social conflicts, DARPP-32, Alternative splicing, RNA-seq

## Abstract

**Background:**

Medium spiny neurons (MSNs) comprise the main body (95% in mouse) of the dorsal striatum neurons and represent dopaminoceptive GABAergic neurons. The cAMP (cyclic Adenosine MonoPhosphate)—mediated cascade of excitation and inhibition responses observed in MSN intracellular signal transduction is crucial for neuroscience research due to its involvement in the motor and behavioral functions. In particular, all types of addictions are related to MSNs. Shedding the light on the mechanics of the above-mentioned cascade is of primary importance for this research domain.

**Results:**

A mouse model of chronic social conflicts in daily agonistic interactions was used to analyze dorsal striatum neurons genes implicated in cAMP-mediated phosphorylation activation pathways specific for MSNs. Based on expression correlation analysis, we succeeded in dissecting Drd1- and Drd2-dopaminoceptive neurons (D1 and D2, correspondingly) gene pathways. We also found that D1 neurons genes clustering are split into two oppositely correlated states, passive and active ones, the latter apparently corresponding to D1 firing stage upon protein kinase A (PKA) activation.

We observed that under defeat stress in chronic social conflicts the loser mice manifest overall depression of dopamine-mediated MSNs activity resulting in previously reported reduced motor activity, while the aggressive mice with positive fighting experience (aggressive mice) feature an increase in both D1-active phase and D2 MSNs genes expression leading to hyperactive behavior pattern corresponded by us before.

Based on the alternative transcript isoforms expression analysis, it was assumed that many genes (*Drd1, Adora1, Pde10, Ppp1r1b, Gnal*), specifically those in D1 neurons, apparently remain transcriptionally repressed via the reversible mechanism of promoter CpG island silencing, resulting in alternative promoter usage following profound reduction in their expression rate.

**Conclusion:**

Based on the animal stress model dorsal striatum pooled tissue RNA-Seq data restricted to cAMP related genes subset we elucidated MSNs steady states exhaustive projection for the first time. We correspond the existence of D1 active state not explicitly outlined before, and connected with dynamic dopamine neurotransmission cycles. Consequently, we were also able to indicate an oscillated postsynaptic dopamine vs glutamate action pattern in the course of the neurotransmission cycles.

## Background

The dorsal striatum is responsible for the regulation of motor activity and stereotypical behaviors and is also potentially involved in a variety of cognitive, reward and social hierarchy processes [1–4]. Through afferent and efferent projections to associative, motor and sensorimotor cortical areas and other brain structures, the dorsal striatum participates in the initiation and execution of movements, as well as in the regulation of muscle tone [5, 6].

Medium spiny neurons (MSNs) represent 95% of neuron population within the dorsal striatum in mice [7]. Notably, MSNs are GABAergic neurons that also have dopamine/glutamate receptors in postsynaptic dendrites. The phosphorylation cascades throughout the dorsal striatum neurons play a central role in motion and emotion signals transduction [8]. In particular, striatonigral “direct” and striatopallidal “indirect” pathways represent opposite excitatory and inhibitory signal transmissions, respectively, depending on MSN dopamine receptor types: Drd1- and Drd2-dopaminoceptive neurons (D1 and D2, correspondingly) gene pathways, D1-dopaminoceptive, or D2-dopaminoceptive [9, 10].

Reciprocal protein phosphorylation/dephosphorylation cascades constitute a major regulatory mechanism of intracellular signal transduction. Its functioning in MSNs, where the corresponding gene pathways are highly expressed and coordinated, manifests a vivid illustration of the process. The networks comprise the pathways mediated by PKA and Cdk5 kinases for cAMP activated kinases, Mapk2-4 for mitogen-activated kinases, serine/threonine phosphatases PP1, PP2A, PP2B and two tyrosine phosphatases Ptpn5, Ptpn7 [8].

Due to the specifics of (de)phosphorylation machinery regulation, multiple inhibitor subunits are recruited in the phosphatase complex formation. In particular, protein phosphatase 1 (PP1) represented by three catalytic subunits alpha, beta, gamma, encoded by *Ppp1ca, Ppp1cb, Ppp1cc* genes*,* can associate with more than 100 inhibitor subunits [11], while PP2A (2 catalytic subunits) inhibitors are represented by 15 + distinct subunits, and PP2B (3 catalytic subunits) is regulated by four inhibitor subunit genes. However, it was found that only one inhibitor can bind the catalytic core at a time via the same catalytic site [12]. Thus, the repressor subunits play a pivotal role in regulating phosphatases in a tissue and stage specific manner.

Since protein phosphatases act in a wide range of cell types and are commonly associated with deactivation and ubiquitylation of proteins [13], *Ppp1r1b* encoding PP1 inhibitor subunit DARPP-32 was underlined as one of the rare neurospecific genes expressed at very high rates specifically in dorsal striatum MSNs and playing a crucial role in the ‘orchestration’ of neurotransmission [14]. *Ppp1r1b* is expressed in the upper bound of the expression range for dorsal striatum-related protein-encoding genes and is implicated as an ultimate factor of MSNs phosphorylation kinetics regulation [15]. It has been proven to be involved in many pathophysiological processes [16–18]. Notably, this gene from the family of protein phosphatase inhibitors solely is directly associated with aggressive behavior as previous studies have revealed [19].

In D1 MSNs cAMPs activate PKA which phosphorylates DARPP-32 on Thr34, transforming it into PP1 inhibitor [20–22]. Calcineurin (PP2B; its catalytic subunit expressed at the highest rate is encoded by *Ppp3ca*), is also expressed in D1 neurons, dephosphorylates DARPP-32 at Thr34, mediating DARPP-32 phosphorylated homeostasis state, in particular turning it off upon signal abrogation. Conversely, Cdk5, which is activated in D2-dopaminoceptive MSNs by Ca2 + provided by AMPA/NMDA receptors, phosphorylates DARPP-32 at Thr75, turning DARPP-32 into PKA inhibitor [23]. The pathway is being regularly updated, and published elsewhere [15]. Besides, MSNs express dorsal striatum specific Tyrosine phosphatase STEP (encoded by *Ptpn5*), which functions alternatively to serine/threonine phosphatases [24].

In the current study we used a mouse chronic social conflicts model allowing to receive male mice with different social experience (aggressive and defeated) in daily agonistic interactions and different motor activity (hyperactivity and total immobility) extensively presented in previous publications since 1991 [25, 26] to analyze the involvement of dorsal striatum neurons in cAMP-mediated phosphorylation activation pathways specific for MSNs. We aimed at assessing the steady states of the D1/D2 neurons based on RNA-Seq expression profiling by considering the reported genes involved in phosphorylation kinetics in MSNs.

## Results

### Compilation of gene sets

To gain fuller insight into the D1/D2 MSNs we retrieved the annotated genes implicated in PP1-regulated phosphorylation pathways in MSNs based on available literature [14, 23, 27–29]. The compiled core gene set comprising cAMP-mediated dopamine response genes, expanded with NMDA glutamate receptor subunits *Grina*, *Grin1-Grin2* and MAP kinases is presented in Table 1. Their expression profiles are presented in Additional file 1: Table S1. We used *Cdk5r1,* neuron-specific activator of cyclin dependent kinase 5 (CDK5) p35 subunit, as a CDK5 activation marker. Three major serine-/threonine-specific phosphatases involved in MSN cascades are PP1, PP2A and PP2B. The PP1 catalytic core comprises 3 subunits (encoded by *Ppp1ca, Ppp1cb, Ppp1cc*), PP2A is represented by 2 catalytic subunits (*Ppp2ca* and *Ppp2cb*), and PP2B includes 3 subunits (*Ppp3ca*, *Ppp3cb, Ppp3cc*). For all three phosphatases the ‘alpha’ subunit exhibits the highest levels of expression (Additional file 1: Table S2), so these subunits were used as the major phosphatase gene markers, except for PP1 (all subunits were considered).

**Table 1:** Core genes set of cAMP-mediated dopamine response involved in the *Ppp1r1b* mediated phosphorylation cycles expanded with NMDA glutamate receptor set (*Grina, Grin1-Grin2*) and Map kinases genes

Gene symbol	Protein name	Description	STR–specific^a^
*Adcy5*	AC5	Adenylate cyclase 5	Yes
*Adora1*	A1A	Adenosine A1 receptor	No
*Adora2a*	A2A	Adenosine A2a receptor	Yes
*Cdk5r1*	CDK5	Cyclin-dependent kinase 5, regulatory subunit 1 (p35)	No
*Drd1*	D1R	Dopamine receptor D1	Yes
*Drd2*	D2R	Dopamine receptor D2	Yes
*Gnai2*	Gi/o	Guanine nucleotide binding protein (G protein), alpha inhibiting 2	No
*Gnal*	Golf	Guanine nucleotide binding protein, alpha stimulating, olfactory type	Yes
*Gpr88*	STRG	G-protein coupled receptor 88	Yes
*Grin1*	NMDA1	Glutamate receptor, ionotropic, NMDA1 (zeta 1)	No
*Grin2a*	NMDA2A	Glutamate receptor, ionotropic, NMDA2A (epsilon 1)	No
*Grin2b*	NMDA2B	Glutamate receptor, ionotropic, NMDA2B (epsilon 2)	No
*Grin2c*	NMDA2C	Glutamate receptor, ionotropic, NMDA2C (epsilon 3)	No
*Grin2d*	NMDA2D	Glutamate receptor, ionotropic, NMDA2D (epsilon 4)	No
*Grina*	NMDARA1	Glutamate receptor, ionotropic, N-methyl D-aspartate-associated protein 1 (glutamate binding)	No
*Map2k1*	ERK1	Mitogen-activated protein kinase kinase 1	No
*Map2k5*	ERK5	Mitogen-activated protein kinase kinase 5	No
*Mapk1*	ERK2	Mitogen-activated protein kinase 1	No
*Mapk3*	ERK1	Mitogen-activated protein kinase 3	No
*Mapk7*	ERK5	Mitogen-activated protein kinase 7	No
*Pde10a*	ADSD2	Phosphodiesterase 10A	Yes
*Pdyn*	PPD	Prodynorphin	No
*Penk*	PPE	Preproenkephalin	Yes
*Ppp1ca*	PP1	Protein phosphatase 1, catalytic subunit, alpha isoform	No
*Ppp1cb*		protein phosphatase 1, catalytic subunit, beta isoform	No
*Ppp1cc*		Protein phosphatase 1, catalytic subunit, gamma isoform	No
*Ppp1r1b*	Darpp32	Protein phosphatase 1, regulatory (inhibitor) subunit 1B	Yes
*Ppp2ca*	PP2A	Protein phosphatase 2 (formerly 2A), catalytic subunit, alpha isoform	No
*Ppp3ca*	PP2B	Protein phosphatase 3, catalytic subunit, alpha isoform (Calcineurin)	No
*Prkaca*	PKA	Protein kinase, cAMP dependent, catalytic, alpha	No
*Ptpn5*	STEP	Protein tyrosine phosphatase, non-receptor type 5	Yes
*Tac1*	PPTA	Tachykinin 1	Yes
*Tac2*	PPTB	Tachykinin 2	No

^a^‘Striatum (STR) -specific’ column indicates genes maintaining striatum specific expression preference

### Gene expression analysis

**Fig. 1:**
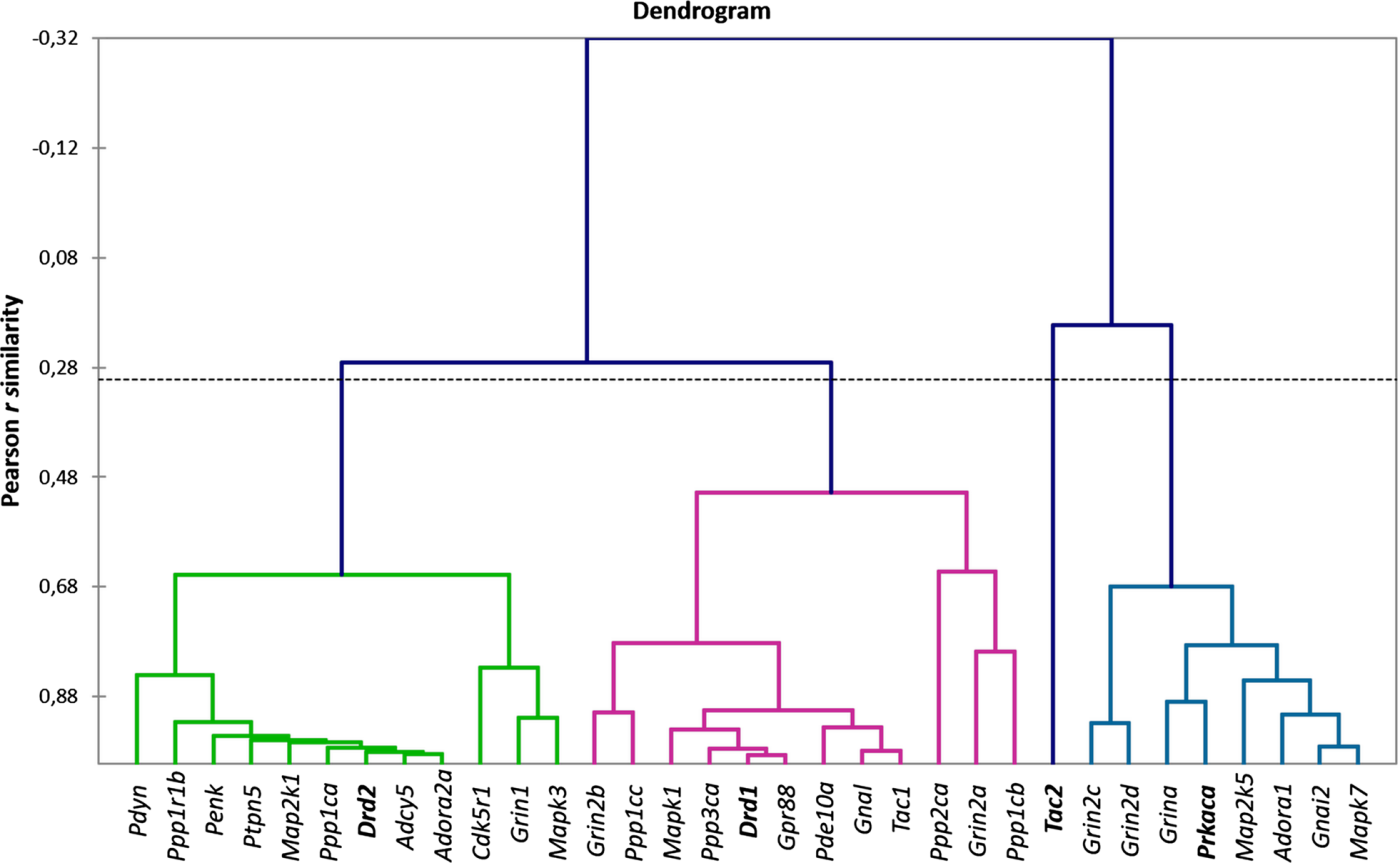
AHC analysis reveals 4 consequent clusters of **a** D2-associated genes (green); **b** D1-associated passive state genes (red); **c** D1-associated active state genes (blue). Single gene corresponding to DA depletion is represented by *Tac2* expression. Cluster—specific marker genes are outlined with bold type

We clustered the core dopamine response cAMP-mediated genes (Table 1) by Agglomerative Hierarchical Cluster (AHC) analysis (Fig. 1) and observed 4 distinct clusters. Strikingly, each of the clusters corresponds to a specific signal transduction cascade observed in cAMP-mediated response to dopamine, which can be ascribed to the key genes. In particular, there is a cluster comprising dopamine *Drd2* receptor distinct from one comprising *Drd1* receptor. Another cluster marks PKA-phosphorylation cascade *(Prkaca)*, and the fourth cluster represented by a single gene (*Tac2*) indicates the absence of dopamine signaling.

**Fig. 2:**
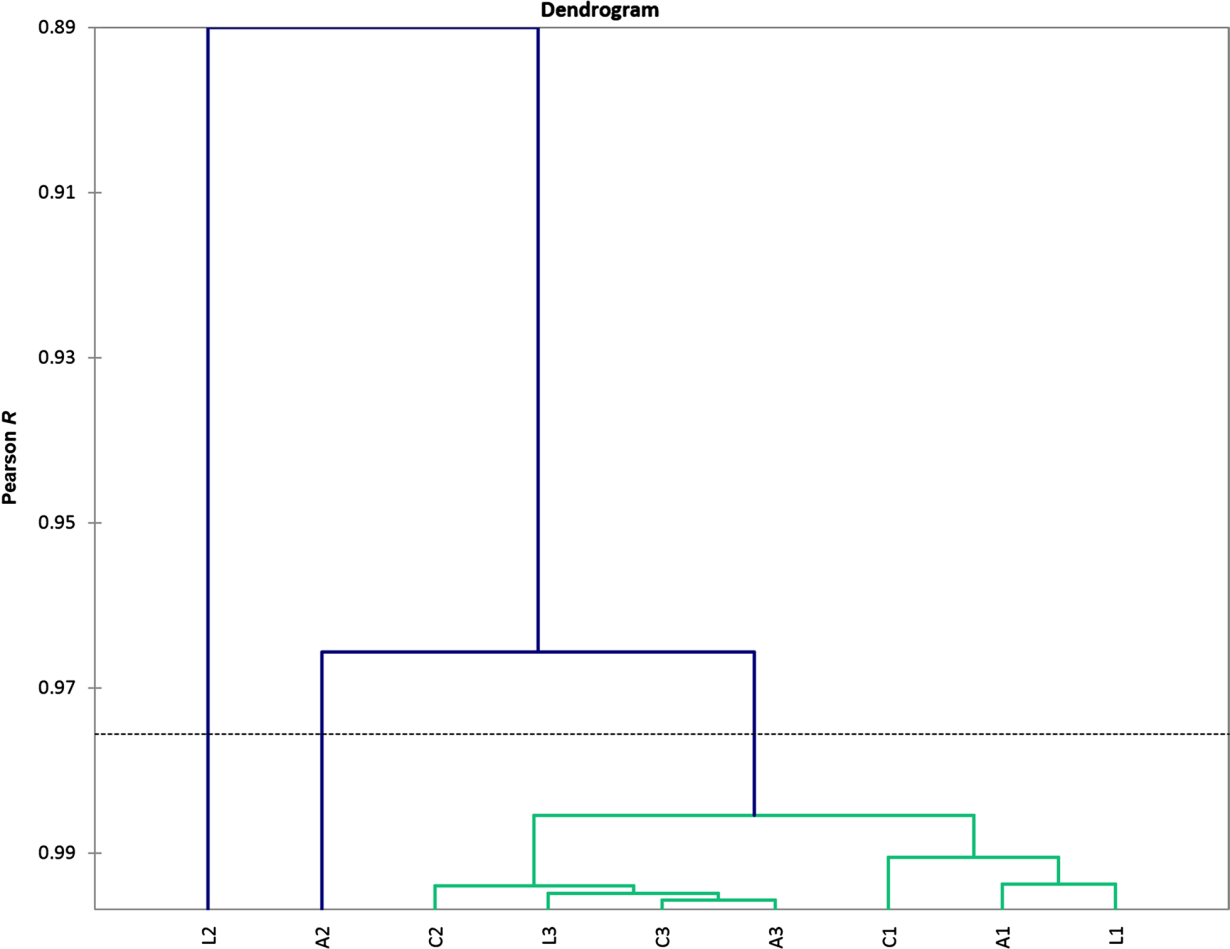
Agglomerative hierarchical clustering of samples (*A* aggressive, *L* loser; *C* control) based on 33 reference genes expression profiles (Additional file 1: Table S1) identified samples L2 and A2 as the most distinct ones in genes expression profiles pattern

Based on the same gene set profiles (Table 1; Additional file 1: Table S1) we also performed AHC procedure samples wise, which revealed rather conservative overall variation rate across the samples, except for two outliers: L2 and A2 (Fig. 2). After careful consideration of L2 and A2 samples we found that the gene expression profiles in these samples didn’t emerge due to spurious technical outbreaks, but manifest highly coordinated events genes expression wise, thus representing the genuine cAMP – mediated genes states distinct from the main group.

**Fig. 3:**
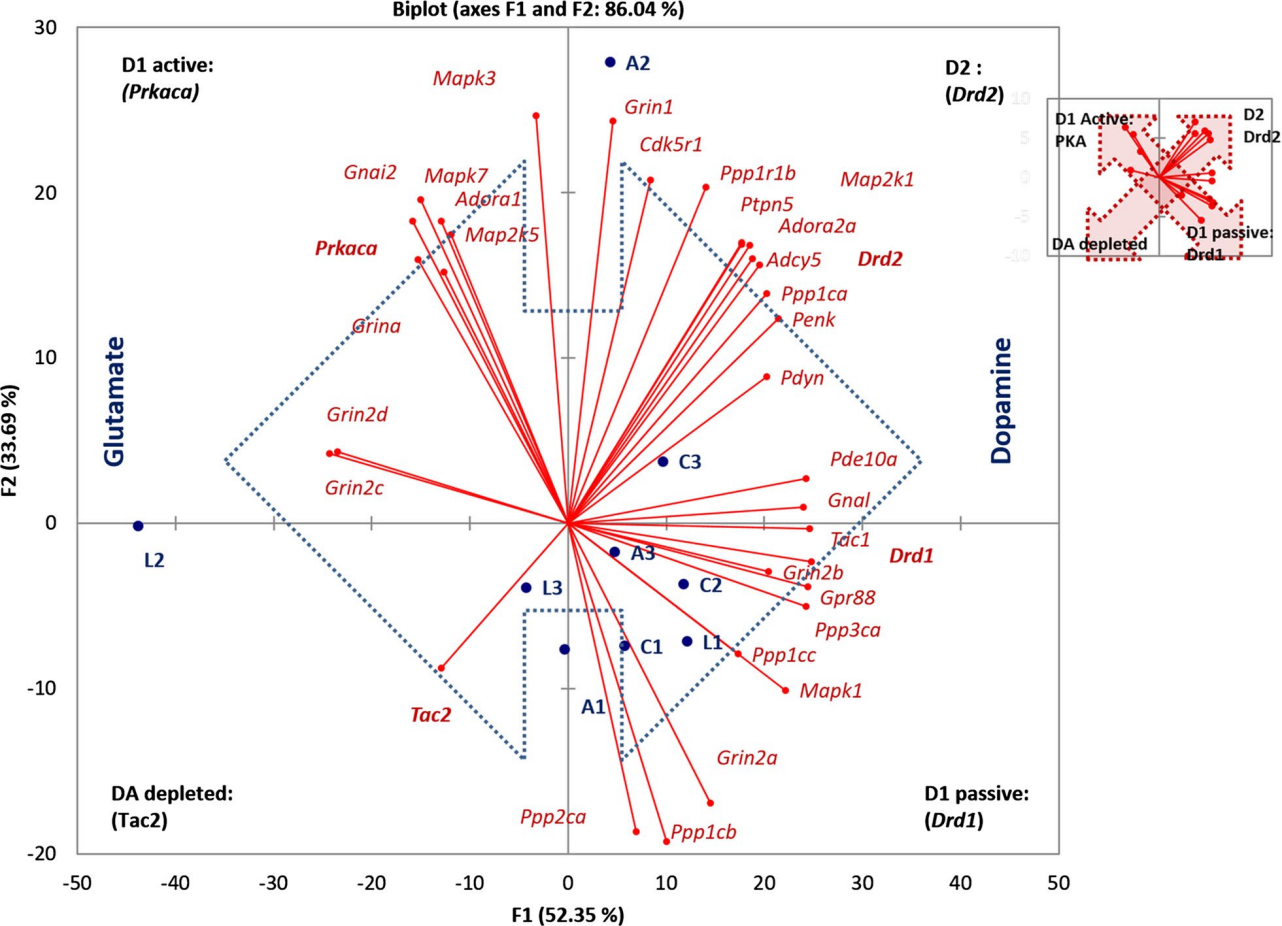
Four major clusters correspondent to each quadrant elucidated by PCA based on 33 reference genes expression profiles (Table 1). Quadrants are denoted with corresponding labels (D1/D2/DA depleted) along with marker gene names. Inserted is a scheme of antagonistic clusters: the arrows depict alternative states of D1 MSN, and D2/DA-depleted states. Blue dotted arrow indicates antagonistic gradient of dopamine and glutamate according to the corresponding receptors distribution. Inserted (small figure) is a scheme of antagonistic clusters: the arrows depict alternative states of D1 MSN, and D2/DA-depleted states. Cluster—specific marker genes are outlined with bold type. Denotations: C1, C2, C3—control; A1, A2, A3—aggressive mice; L1, L2, L3—losers, defeated mice

To gain an expanded view on the clusters elucidated by AHC (Fig. 1) we performed the Principal Components Analysis (PCA) on the same core genes set (Fig. 3). It has been confirmed that the gene clusters and samples are highly synchronized (86% variation coverage overall for two top components), each corresponding to a particular neuron type (D1/D2) according to their annotation in publications. Also, as follows from Fig. 3, we can observe antagonistic clusters of the D1 neurons indicating its stable rather than passive state followed by a short firing time span upon activation represented by D1 active state as shown in [29]. The biplot of the PCA analysis is shown in Fig. 3. According to the marker genes within the clusters, they were designated as D2 cluster, D1 passive, D1 active and DA depletion.

### Regulation of *Ppp1r1b* expression

It is known that in phosphorylated state DARPP-32 effectively inhibits both PP1 alpha (encoded by *Ppp1ca*) [30] and PP1 gamma (encoded by *Ppp1cc*) [31]. As we can see from the plot in Fig. 3*Ppp1r1b* is located in the D2 cluster along with *Ppp1ca* encoding PP1 alpha subunit, which indicates that this cluster is a major expression site. However, another catalytic subunit of PP1, *Ppp1cc,* is located in D1 passive cluster (Fig. 3). Given *Ppp1cc* is committed specifically to D1 passive state (Fig. 3) one may expect its co-expression with *Ppp1r1b* in D1 neurons.

Similar to *Grin1* (major subunit of NMDA) committed to D2 cluster, *Grin2a-d* subunits are distributed across distinct quadrants in Fig. 3: *Grin2a, b* are associated with D1 neurons passive state, while *Grina, Grin2c, d* are located in D1 neurons active state cluster. Thus, multiple subunits complexes allow to envision other genes in the cluster upon PCA grouping.

### Expression rates of dorsal striatum-specific gene clusters as compared to four other brain regions

**Fig. 4:**
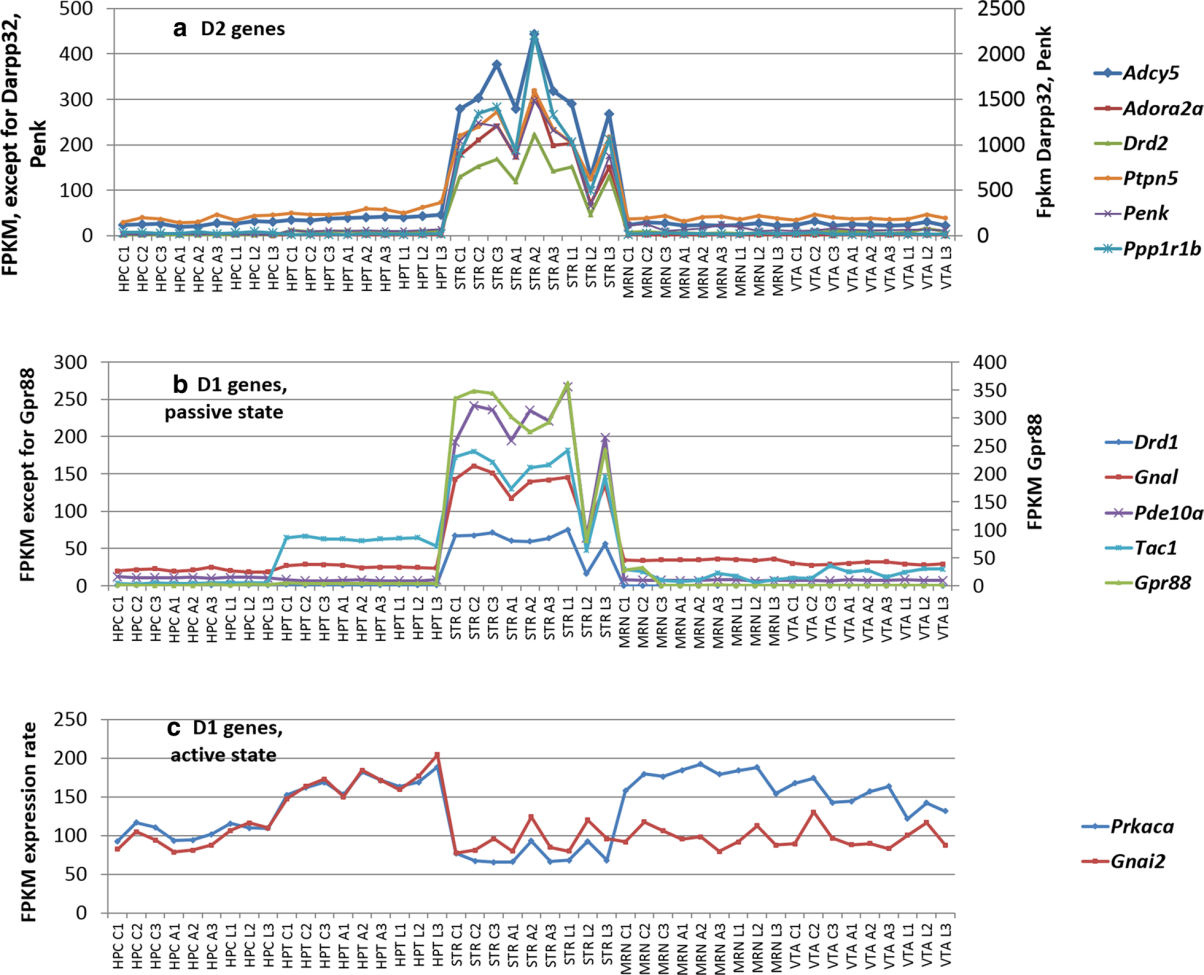
Profiles of D1/D2 dorsal striatum specific genes expression (**a**, **b**), and non (anti) -specific ones in D1-active pathway (**c**) across 5 brain regions. C1, C2, C3—control; A1, A2, A3—aggressive mice; L1, L2, L3—losers, defeated mice. *HPC* Hippocampus, *HPT* Hypothalamus, *STR *Dorsal striatum, *MRN* Midbrain raphe nuclei, *VTA* Ventral tegmental area

Average expression rate of the genes has been analyzed in distinct clusters (described above) across 5 brain regions: hippocampus (HPC), hypothalamus (HPT), dorsal striatum (STR) ventral tegmental area (VTA), midbrain raphe nuclei (MRN). Several genes have been identified as the genes preferentially expressed in the dorsal striatum. Expression profiles of the selected genes are shown in Fig. 4a, b. They happen to be expressed specifically in D2 and D1 passive state clusters. As for D1 active cluster, we identified *Prkaca* (encoding one of the PKA subunits) and *Gnai2* as the genes with the lowest expression levels in the dorsal striatum across 5 brain regions (Fig. 4c). Figure 3 illustrates that the most actively expressed genes are associated with D2 cluster (Fig. 3a). *Ppp1r1b* and *Penk* genes with more than 1000 FPKM units may be considered as the signature, driver genes within D2 cluster. The selected genes in D1 passive cluster also maintain a dorsal striatum-specific expression pattern (Fig. 4b), but their average expression rate was lower than that of D2 cluster genes (Fig. 4a), implying the major role of D2 cluster, and DARPP-32 in particular as the basic maintenance unit of dorsal striatum functioning.

Notably, in the above-mentioned two clusters (D2 and D1 passive), the expression rate of selected genes was significantly higher in the dorsal striatum compared to other four brain regions. On the contrary, assessment of the signature genes (*Prkaca, Gnai2*) of D1-active cluster demonstrated patently decreased expression rates of *Prkaca* and *Gnai2* genes as compared to other brain regions (Fig. 4c), implying a short expression time span upon firing as one of the reasons.

Additionally, we present the differential expression analysis of the abovementioned genes set in Additional file 1: Table S3. We found that aggressive group didn’t differ from controls in any of the genes considered. On the contrary, loser mice group indicated significant difference both in D1 and D2 clusters (Additional file 1: Table S3).

### Alternative splicing analysis

**Fig. 5:**
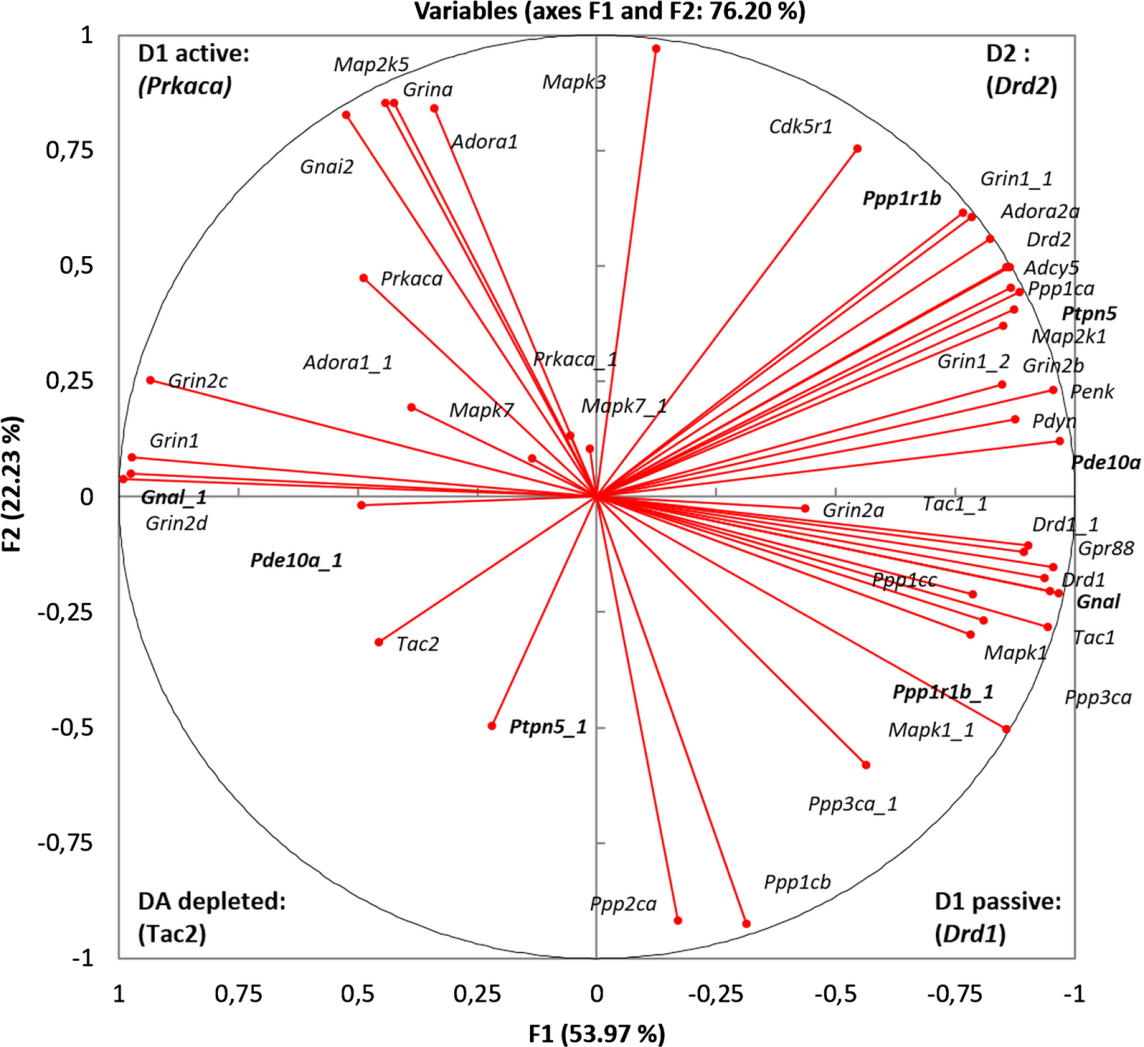
Distribution of splice isoforms across D1/D2 clusters based on 83 transcripts (Additional file 1: Table S2). In bold are splice isoforms that are negatively correlated, or significantly differed (bold italic) in their location. Transcript denotations are listed in Additional file 1: Table S3

We retrieved 13 additional minor splice isoforms for 12 genes from our set of 33 core genes (Additional file 1: Table S4). After that we performed PCA for the Pearson pairwise correlation matrix. A circular plot for 46 resulting transcripts is shown in Fig. 4. Most splicing isoforms, namely for genes *Prkaca, Adora1, Mapk7, Mapk1, Tac1, Drd1, Penk, Ppp3ca* (Fig. 5), exhibit concordant expression patterns in the same clusters or close to them. However, we identified negatively correlated, mutually exclusive splicing isoforms for three pairs of gene transcripts: *Pde10-Pde10_1, Ptpn5-Ptpn5_1, Gnal-Gnal_1* (Fig. 5). Notably, *Pde10a_1, Ptpn5_1* and *Gnal_1* represent minor long isoforms (Additional file 2: Figure. S1a–c) with negligible expression rates (4–100-fold lower; Additional file 1: Table S3) as compared to corresponding major transcripts. According to the results shown in Fig. 5 one may suggest that the transition from D1 passive to D1 active state is accompanied by the switch between transcription patterns of *Pde10* and *Gnal* genes. Also, the transition from D2 state to dopamine depleted state is signified by the switch between transcription patterns of *Ptpn5*.

The *Ppp1r1b* minor splice variant (*t-DARPP* or *Ppp1r1b*_1 in Additional file 1: Table S3) was found to be specific to D1 MSNs in a passive mode and to exhibit fivefold reduction in expression level as compared to that of major D2 cluster-associated transcript (Fig. 5). The truncated isoform of *Ppp1r1b* (*t-DARPP;* NM_001313970; Additional file 2: Fig. S2a) [32] lacking 37aa at *N*-terminal makes T34 inaccessible to phosphorylation by PKA as well as prevents its overall binding to PP1 while retaining the ability to inhibit PKA upon T75 phosphorylation.

### Role of CpG islands in alternative transcription

The genes listed above (*Ppp1r1b, Ptpn5, Pde10a* and *Gnal*) maintain CpG islands associated with promoters. Remarkably, upon transition of D1 passive to D1 active state and consequent gene networks rearrangement, some of the alternative long isoforms start being transcribed from its closest distal CpG islands (Additional file 1: Figures S1 and S2). The alteration is witnessed by negative correlation of the splicing isoforms. This is the case with *Pde10a*, *Gnal* genes (Additional file 2: Figure S1), implying that the mechanism of transcription pattern switching is based on the inhibition of proximal promoter CpG island of the major isoform transcript. Two other genes, *Ppp1r1b* and *Ptpn5,* maintain CpG promoter in the major isoform but with non-CpG mediated alternative transcription start site (Additional file 2: Figures S1 and S2). As the above-mentioned genes are involved in D1 passive/active state dynamics, CpG island inhibition process may support the propensity for rapid reciprocity upon D1 active state abrogation and the restoration of the genes’ default isoforms.

## Discussion

The dynamics of MSN related cAMP-mediated dopamine response, even though intensively studied, needs to be elaborated further [29]. After the emergence of the single cell transcriptome protocol, in the study by Gokce et al. [33] and in a range of subsequent publications dedicated to single cell analysis, the high resolution taxonomy of D1/D2 neurons and glial cells in the dorsal striatum was depicted. Still, the current study showed that the pooled tissue analysis may yield a unique insight based on the consideration of specific genes subset.

We pursued the task of elucidating the phase portrait of dopamine cAMP-mediated response based on specific genes subset expression profiles to assess the asymptotic steady states of MSNs in the dorsal striatum, taking into account the fact that the dorsal striatum cell content is highly homogeneous. It was postulated that cell identity is determined by specific gene expression signature as it is in single cell analysis. In our work we found four distinct gene clusters, namely: one corresponding to D2 MSNs, another one—to dopamine depleted state, and two clusters—to opposite D1 passive/active states. Single-cell results on striatum neurons [33] largely corroborate D1/D2 marker genes identified in this study. The analyzed clusters feature the following properties:

1. D2 cluster. While it is known that *Ppp1r1b is* expressed both in D1- and D2 MSNs, in the current study the expression rate of *Ppp1r1b* proved to be strongly correlated with those for *Cdk5 (p35)* along with *Drd2, Adora2* and *Penk,* implying that a major fraction of *Ppp1r1b* expression rate in the dorsal striatum has to be ascribed to D2 MSNs, possibly due to neuroendocrine response involvement. Notably, phosphorylation of DARPP-32 and D1R by CDK5 has been reported previously [34]. Expression of *Ppp2ca* and *Ppp3ca* genes encoding the catalytic subunits for specific serine-/threonine- protein phosphatases PP2A, PP2B was not found in this cluster by the clustering analysis (Figs. 1 and 3). Instead, the previously reported tyrosine phosphatase STEP encoded by *Ptpn5* is intensely expressed in the neurons of this type [1]. In D2 MSNs *Adora2A-Drd2-Adcy5* genes constitute a highly interlinked heteromeric transmembrane receptor complex elucidated recently, featuring D2-MSNs [35, 36].
2. D1 cluster (passive phase). *Ppp2ca* (PP2A) and *Ppp3ca* (PP2B*)* genes were found to be specifically expressed in D1-neurons, apparently exemplifying dephosphorylation of DARPP-32 upon signal abrogation, thereby maintaining homeostasis of phosphorylated DARPP-32 proteins at a certain level mediated by AMPA/NMDA Ca2 + input rate. Also, experimental findings [37] that *Golf*, *Gpr88* are the major G-proteins facilitating preferential maintenance of *Drd1* receptors in MSNs found confirmation in our study. As mentioned earlier [38], preprotachykinin A (*Tac1) e*xpression is highly correlated with that of D1 receptor. As follows from correlation analysis, ERK2 (*Mapk1)* kinase is also involved in maintaining D1 MSN passive phase and was shown to be associated with D1 neurons [39]. Notably, the truncated form of *Ppp1r1b* (*t-DARPP*) was previously reported for several brain structures [40], but it is the first time that *t-DARPP* has been mapped to MSNs network D1 cluster.
3. D1 cluster (active phase). We observed coordinated expression of *Prkaca, Grina* and *Adora1a* genes in active D1 MSNs. Their relation to D1 neurons was reported previously [14], along with *Grin1*, *Map2k1*/*Mapk3* (*ERK1*) and *Map2k5*/*Mapk7* (*ERK5*) genes [41], which were also localized in this cluster in our study.
4. The fourth cluster was represented by single gene, *Tac2* (Fig. 3) which maintains minimal striatal FPKM level of 0.5–1 units observed in the current study. Preprotachykinin B (*Tac2*) is known to be expressed in only 5% of dorsal striatum non-MSN neurons [42]. It implies that *Tac2* gene may be chosen as a signature indicator of non-D1/D2 MSN neuron type lacking specific dopamine response, thus indicating the dopamine-depleted state.

### Glutamate vs dopamine gradient in MSN states

As it is known, there are multiple other neurotransmitters besides dopamine that regulate the excitability of dopaminoceptive neurons: glutamate, GABA, opiates, and adenosine, which are involved in signaling through DARPP-32 in MSNs [28]. In particular, in our previous studies the expression rates of catecholaminergic, glutamatergic and GABAergic receptors in the dorsal striatum were shown to be significantly altered as compared to the control group, in reverse directions for the defeated and aggressive mice [25, 26]. Herein we confined our attention to dopamine and also glutamate (due to its relevance to cAMP phosphorylation) signaling in MSNs.

While the variation among the groups of experimental animals yielded no particular group-specific clustering (Figs. 2and 3), the observed samples obtained from defeated mice (L2, L3) exhibit a bias toward the dopamine-depleted quadrant (Fig. 3). Aggressive mice (A2- winner) display an increase in both D1 and D2 expression (Fig. 3). Other samples tend to maintain passive D1 phase and moderate D2 MSNs genes expression.

Notably, a behavior animal model employed in the study was potent in significantly augmenting the physiological states variation of samples, this way uncovering dopamine-depleted/glutamate rich area specific states (D1 active, DA depleted). Graphically it is represented in Fig. 3 by blue arrow underlining dopamine vs glutamate gradient based on *Drd1/Drd2* vs *Grin1/Grin2* receptor genes distribution. Specific combinatorial usage of *NMDA1* receptor subunits (*Grin2a*, *b, c, d*) across various clusters (Figs. 1 and 3) was observed in ensemble with *Grin1* isoforms preferences: major *Grin1* isoform was prevailed in PKA-mediated D1 active cluster*,* while *Grin1_1, Grin1_2* feature specifically D2 cluster (Fig. 5; Additional file 1: Table 4).

Intriguingly, both loser (L2) and aggressive (A2) mice manifest elevated glutamate neurotransmission (Fig. 3; left half of the plot), but for completely different reasons: the aggressive one apparently maintains a high frequency of D1 neurons firing, associated with PKA and calcium influx activation maintained by increased *NMDA1* subunits expression rate. It goes along with high dopamine uptake background in aggressive mice (according to Drd1/Drd2 expression rates). Conversely, an increased glutamate transmission in L2 loser mousee signifies the response on the lack of overall dopamine intake in their dorsal striatum.

The ‘up’ and ‘down’ states of D1 neurons were reported previously [8, 43]: the ‘firing’ of D1 neurons upon glutamate input is reported to maintain peak-like induction of voltage increase spanning about 1 s [43] incomparable with the down state span for the major time lapse. It is also confirmed by Fast Spiking (FS) interneurons preferentially targeting Direct (D1) pathway using AMPA receptors, while D2 pathway is targeted by persistent low-threshold spiking (PLTS) interneurons, and NMDA receptors [29, 44].

### D1 MSN oscillating states employ alternative transcripts switching

When analyzing alternatively spliced transcripts, we found a set of genes employing alternative promoter usage (Additional file 2: Figure S1). Most of them are associated in particular with D1 neurons (*Drd1, Gnal, Ppp1r1b, Pde10a;* Additional file 1: Table S3; Additional file 2: Figures S1 and S2), implying a possible mechanism for gene/transcripts switching through reversible blocking of CpG island promoters (Additional file 1: Table S3; Additional file 2: Figures. S1 and S2). It is worth noting that the minor isoform is usually longer (Additional file 1: Table S3), implying the inhibition of proximal CpG island as reported earlier [45]. As the switching between D1 active – passive states is of oscillatory nature, CpG islands are apparently not modified by DNA methylation as was the case in [45], nor are alternative histone modifications [46], since both isoforms are present in D1 neurons, so it might be some repressive transcription factor not yet assigned. It is also intriguing that the majority of the isoforms encode alternative functional proteins, which are expressed in D1 active state, but usually at significantly lower rates (Additional file 1: Table S3).

### MSN dynamics schema

The performed correlation analysis was based on the previous experimental evidence that the synaptic genes network expression is highly coordinated and specific [47, 48]. Our attempt to shed a light on such intricate genes networks was based on the presumption that proteins phosphorylation/ dephosphorylation cascades provide relevant feedback for genes expression rate, which was found to be well-grounded in our study.

**Fig. 6:**
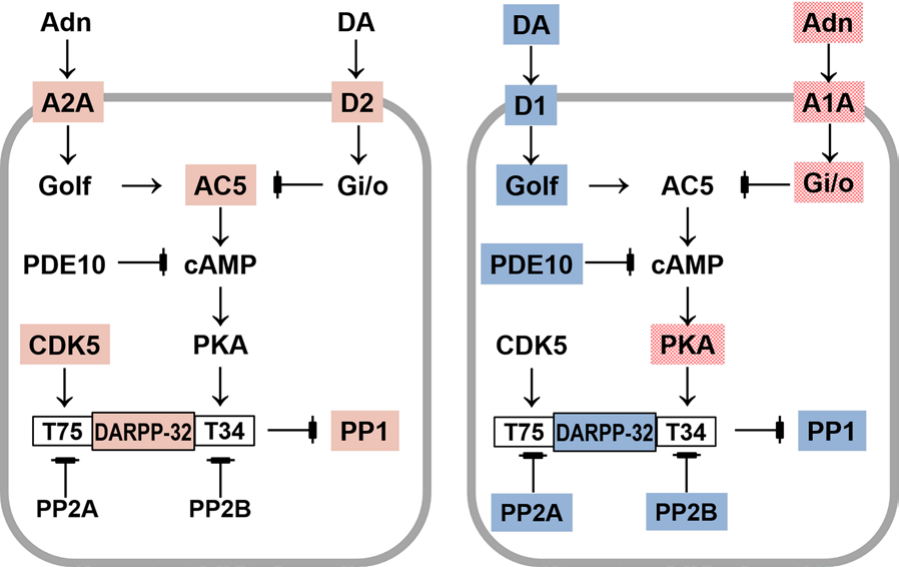
Two types of MSNs, D2 (**a**) and D1 (**b**) are represented by the same cAMP-centered gene cascades. Colored are correlated gene clusters observed in our study (3 clusters; Fig. 1). **a** Stable D2-A2A-Golf-Gi/o complex features preferential inhibition of AC5→cAMP synthesis upon DA (dopamine) signal, though stimulants can invoke it [35, 36]. Genes encoding proteins shaded red are coordinately regulated in D2 neurons as observed in our RNA-Seq data (Fig. 1). Genes encoding uncolored proteins are absent in the D2 cluster (Fig. 1), implying they may be present with minor expression ranges and are not involved in coordinated variation. **b** Oscillating passive-active cascades in D1 neurons. Genes encoding proteins of the same color manifest correlated clusters in our data presented in Fig. 2. Blue (D1 passive state) vs Red (D1 active state) clusters are antagonistic ones according to AHC and PCA analyses (Figs. 1 and  3). Genes encoding uncolored proteins are absent in the D1 cluster (Fig. 1), implying they may be present with minor expression ranges and are not involved in coordinated variation

The expression profiling of the genes involved in D1/D2 MSNs phosphorylation cascades has proved informative, and concordant with the current knowledge on corresponding gene pathways in these neurons. In particular, we provided an illustration of events identified in the study by Fig. 6a, b. The proteins ascribed in our study to the genes from D1/D2 clusters are marked with color. So, the schematics proposed by [29] may be helpful for gaining the expression-based insight into the specifics of MSNs functioning.

Thus, D1 MSN excitation kinetics exerts the major impact on the D1/D2 cascades dynamics as follows from D1-split states observations (featuring PKA cascade activation), while D2 neurons manifest the strongest coordinated background expression of the target genes, including postsynaptic *Drd2- Adora2- Adcy5* receptor complex, *Ppp1r1b*, and *Penk* (Fig. 1). In particular, *Drd2* expression rate in the dorsal striatum is twofold higher than that of *Drd1* (Additional file 1: Table S1)*.* High *Penk* expression rate observed in D2 neurons (Additional file 1: Table S1) pinpoints D2 MSNs entity as a major neuroendocrine response body in dorsal striatum previously reported in [49–51].

## Conclusion

Overall, the study outlines three key points. First, using animal stress model allowed elucidating the oscillating states in D1 neurons within cAMP mediated genes subset, featuring two distinct genes pathways: Drd1–mediated and PKA-mediated ones. Second, we partly elaborate on the molecular mechanics of D1 oscillating states by analyzing the alternative transcripts. Third, we report the extreme aggressive and depressive states in striatum genes cascades which feature dopamine – glutamate neurotransmission interplay specifics in cAMP related gene expression dynamics.

Thus, the study has two basic advantages/reasons: a) employing the stress animal model has significantly expanded the variation of the samples in gene expression profiles spectra; b) using pooled tissue samples allowed assessing the steady state expression landscape of cAMP mediated genes. As a further move in this direction, we may try to use other specific genes subset to elaborate on a range of questions that arise, in particular: what is the exact source of glutamate neurotransmission? Is there any endogenous glutamate expression in loser mice striatum? Is there a way to find the best phenotypic marker for the species with deviant gene expression profiles in striatum, so we can a priori know that?

## Methods

### Experimental animals

Adult C57BL/6 male mice were purchased from Animal Breeding Facility, Branch of Institute of Bioorganic Chemistry of the RAS (ABF BIBCh, RAS) (Pushchino, Moscow region). The housing of animals conformed the standard conditions, namely: 12:12 h light/dark regime starting at 8:00 am, at a constant temperature of 22 ± 2C The food in pellets and the water were available ad libitum. Mice were weaned at three weeks of age and housed in groups of 8–10 per standard plastic cage. The age of animals at the time of experiment engagement was 10–12 weeks old.

### Ethical statement

All procedures were in compliance with the European Communities Council Directive 210/63/EU of September 22, 2010. The protocol for the studies was approved by Scientific Council No 9 of the Institute of Cytology and Genetics SD RAS of March, 24, 2010, N 613 (Novosibirsk, https://spf.bionet.nsc.ru/).

## Experimental procedures

### Generation of alternative social experiences under daily agonistic interactions in male mice

Prolonged negative and positive social experience, social defeats and wins, in male mice were induced by daily agonistic interactions [52, 53]. Pairs of weight-matched animals were each placed in a steel cage (14 × 28 x 10 cm) bisected by a perforated transparent partition allowing the animals to see, hear and smell each other, but preventing physical contact. Before being exposed to encounters, the animals were left at rest for two or three days adapting to new housing conditions and a sensory contact. Every afternoon (14:00–17:00 p.m. local time) the cage lid was replaced by a diaphanous one, and after 5 min (the period necessary for individuals' activation) the cage partition between individuals was removed for 10 min enabling agonistic interactions. The winning mouse was unambiguously established after two or three physical interactions with the opponent. In particular, the superior mouse would be attacking, biting and chasing opponent who displays only defensive behavior (sideways postures, upright postures, withdrawal, lying on the back or freezing). Aggressive interactions session between males was discontinued by installing the cage partition if the sustained attacks have lasted more than 3 min (in some cases less) preserving the defeated mouse from further attacks. Each defeated mouse (loser) was exposed to the same winner for three days. After the fight session, each loser was placed in an alien cage with an (unfamiliar) winner behind the partition until the next day encounter. On the contrary, the winners were constantly hosted within their original cage. The listed encounter protocol was performed once a day for 20 consequent days. An equal number of the winners and losers were enucleated. The explicit behavioral data on the model has been published in [53, 54].

According to the protocol listed above, we employed three groups of animals in the study: (1) Controls—the mice without a consecutive experience of agonistic interactions; (2) Losers—chronically defeated mice; (3) Winners—chronically aggressive mice. The losers and winners with the most eminent behavioral phenotypes were selected for the transcriptome analysis. Each group comprised three animals in the current study. The winners manifested the highest attacking instances number as well as total attacking time and the shortest latency of the first attack. Aggressive grooming, threats (tail rattling), hostility during 20-day experiment were also manifested. The losers regularly displayed the full submission posture (“on the back”), opponentavoidance and the largest passive defense timespan (freezing, immobility) in the course of agonistic interaction test. Overall, the chronically aggressive mice developed motor hyperactivity, enhanced aggressiveness and stereotypic-like behaviors, while chronically defeated mice manifested low motor activity and depression-like behaviors. We refer the behavioral data details in our model to be explicitly presented in [25, 26, 54].

The control animals and the affected mice were simultaneously decapitated 24 h after the last agonistic interaction. The brain regions were dissected by the same person according to the Allen Mouse Brain Atlas map [https://mouse.brain-map.org/static/atlas]. Biological samples were placed in RNAlater solution (Life Technologies, USA) and stored at −70 °C.

The brain regions selection for the analysis was based on their functions reported elsewhere as implicated in behavior manifestation. They were: the midbrain raphe nuclei (MRN), a multifunctional region of brain containing the body of the serotonergic neurons; the ventral tegmental area (VTA) containing the pericaryons of the dopaminergic neurons, which are widely implicated in brain reward circuitry and are important for motivation, cognition, drug addiction, and emotions relating to several psychiatric disorders; the striatum (STR), which is a mediator of stereotypical behaviors and motor activity, also implicated in cognitive processes; the hippocampus (HPC), a part of the limbic system essential for memory consolidation and storage, playing an distinct role in emotional modulation; the hypothalamus (HPT), which mediates the stress response within Hypothalamic–pituitary axis (HPA), typical for our model.

## RNA-Seq data collection

We submitted the collected brain samples to JSC Genoanalytica (www.genoanalytica.ru, Moscow, Russia) for RNA-Seq routine. mRNA was extracted using a Dynabeads mRNA Purification Kit (Ambion, Thermo Fisher Scientific, Waltham, MA, USA). cDNA libraries were created using the NEBNext mRNA Library PrepReagent Set for Illumina (New England Biolabs, Ipswich, MA USA) according to the manufacturer’s protocol. Illumina HiSeq 2500 System was used sequencing using single (non-paired end) reads of 50 bp length. The target coverage was set to 20 Mio. reads per sample. The resulting reads were aligned against the GRCm38.p3 reference genome using the STAR aligner [55]. *Cuffnorm* software [56] was employed for expression rate assessment in FPKM units and for the alternatively spliced transcripts expression profiles reconstruction. The brain regions were processed for each of 3 animals per group, separately, without technical replicates. Three groups of animals were employed in the study, totaling in 9 distinct samples per brain region.

### Statistical analysis

Principal Component Analysis **(**PCA) was employed using the XLStat statistical package (www.xlstat.com). Pearson product moment correlation matrix for genes expression set in 9 samples total was used as input data for PCA. The same matrix was used for the Agglomerative Hierarchical Clustering (AHC) analysis with XLStat. The agglomeration method implied an unweighted pair-group average; no data centering was employed. We avoided using commonly accepted WGCNA method [57] due to its detrimental impact on clustering density because of limited amount of our genes sample (up to 100 transcripts).

## Supplementary information

**Additional file 1: Table S1.** Encoding and expression profiles of transcripts. **Table S2.** Expression profiles of phosphatase subunit genes. **Table S3.** Encoding and expression profiles of transcripts. Gene symbol denotes the presence of transcripts variation in the names supplied with dash followed by number of (minor expressed) splice isoform.

**Additional file 2: Figure S1.** Mutually exclusive and negatively correlated transcript variants for *Pde10a (a), Gnal (b), and Ptpn5 (c)* maintaining alternative CpG promoters. **Figure S2.** Structure of alternative transcripts of two genes *Ppp1r1b* (DARPP-32) (**a**), *Drd1* (**b**) based on their alternative (non-CpG) promoters.

## Data Availability

The additional statistics of data obtained used to support the findings of this study are available from supplementary files. The raw sequence data for 9 dorsal striatum samples were deposited in ENA archive PRJEB36194.

## Abbreviations

MSN: Medium Spiny Neurons
cAMP: Cyclic Adenosine MonoPhosphate
D1/D2 neurons: drd1/Drd2 Dopaminoceptive neurons
DARPP-32: Dopamine- and cAMP-regulated neuronal phosphoprotein 32 kDa
PCA: Principal component analysis
AHC: Agglomerative hierarchical clustering
FPKM: Fragments per kilobase of transcript per million mapped reads
