# Supplementary material for: Dopamine response gene pathways in dorsal striatum MSNs from a gene expression viewpoint: cAMP-mediated gene networks
Source: BMC Neurosci. 2020 Mar 26;21:12. doi: 10.1186/s12868-020-00560-w (PMC7099774; doi:10.1186/s12868-020-00560-w)
Supplement: Supplementary file 2 — Additional file 2: Figure S1. Mutually exclusive and negatively correlated transcript variants for Pde10a (a), Gnal (b), and Ptpn5 (c) maintaining alternative CpG promoters. Figure S2. Structure of alternative transcripts of two genes Ppp1r1b (DARPP-32) (a), Drd1 (b) based on their alternative (non-CpG) promoters. [file 12868_2020_560_MOESM2_ESM.docx]

| 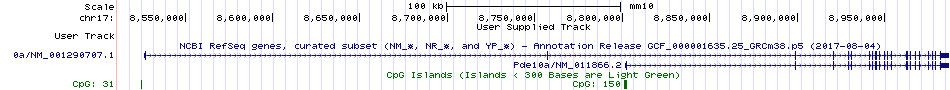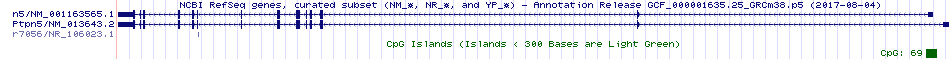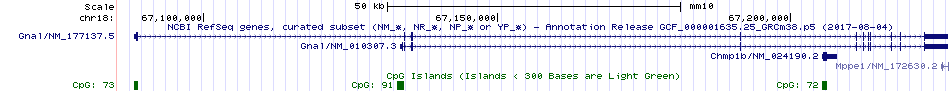 b  c  a  **Supplemental Figure S1.** Mutually exclusive (negatively correlated) splice variants for *Pde10a (a), Gnal (b), and Ptpn5 (c)* schema of the alternative CpG promoters. Corresponding CpG islands are encircled. |
| --- |

| 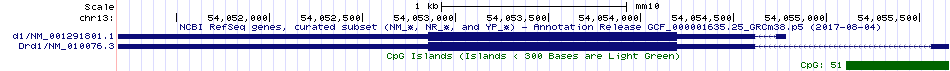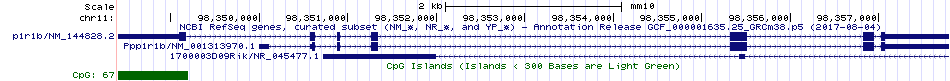 a  b  **Supplemental Figure S2.** Structure of alternative isoforms of two genes *Ppp1r1b* (DARPP-32) (a), *Drd1* (b) based on their alternative (non-CpG) promoters. |
| --- |
